# The Effect of Two Different Cognitive Tests on Gait Parameters during Dual Tasks in Healthy Postmenopausal Women

**DOI:** 10.1155/2016/1205469

**Published:** 2016-02-28

**Authors:** Magdalena Hagner-Derengowska, Krystian Kałużny, Wojciech Hagner, Anna Kałużna, Bartosz Kochański, Alina Borkowska, Jacek Budzyński

**Affiliations:** ^1^Chair of Clinical Neuropsychology, Faculty of Health Sciences, Nicolaus Copernicus University in Toruń, M. Skłodowskiej-Curie 9 Street, 85-094 Bydgoszcz, Poland; ^2^Chair and Clinic of Rehabilitation, Faculty of Health Sciences, Nicolaus Copernicus University in Toruń, M. Skłodowskiej-Curie 9 Street, 85-094 Bydgoszcz, Poland; ^3^Chair of Vascular and Internal Diseases, Faculty of Health Sciences, Nicolaus Copernicus University in Toruń, Ujejskiego 75 Street, 85-168 Bydgoszcz, Poland

## Abstract

*Introduction*. The paper aims to evaluate the influence of two different demanding cognitive tasks on gait parameters using BTS SMART system analysis.* Patients and Methods*. The study comprised 53 postmenopausal women aged 64.5 ± 6.7 years (range: 47–79). For every subject, gait analysis using a BTS SMART system was performed in a dual-task study design under three conditions: (I) while walking only (single task), (II) walking while performing a simultaneous simple cognitive task (SCT) (dual task), and (III) walking while performing a simultaneous complex cognitive task (CCT) (dual task). Time-space parameters of gait pertaining to the length of a single support phase, double support phase, gait speed, step length, step width, and leg swing speed were analyzed.* Results*. Performance of cognitive tests during gait resulted in a statistically significant prolongation of the left (by 7%) and right (by 7%) foot gait cycle, shortening of the length of steps made with the right extremity (by 4%), reduction of speed of swings made with the left (by 11%) and right (by 8%) extremity, and reduction in gait speed (by 6%).* Conclusions*. Performance of cognitive tests during gait changes its individual pattern in relation to the level of the difficulty of the task.

## 1. Introduction

Gait is a complex motor activity involving many musculoskeletal elements. However, although it is the basic mechanism of human movement and is considered one of the vital signs, it is not only an automatic process but also an attention-demanding task [1–4]. Various walking behaviors require a different attentional load [1] and different cognitive tasks performed simultaneously with walking change the gait pattern [5]. The overlapping of motor and cognitive functions occurs every day during regular walking, when walking is accompanied by attention-demanding situations, such as overcoming barriers and obstacles, the use of a mobile phone, responses to changes in the color of traffic lights (stop or go), reactions to instructions, and the sounds of moving cars or horns [6–9]. Disturbances in these complex relationships lead to many clinical conditions, one example of which is motoric cognitive risk (MCR) syndrome, a newly described predementia syndrome characterized by slow gait and cognitive complaints [10]. Diagnosis of this syndrome is recognized as a risk factor for dementia and frailty [11]. Other clinical conditions in the course of which motor-cognitive disorders are observed are as follows: depression [12–14], dementia [15], Parkinson's [16–21] and Alzheimer's diseases [22], and multiple sclerosis [23]. The prevalence of these disturbances is also greater in older people due to age-related reduction in the ability to allocate attention selectively across multiple domains, more pronounced dual-task interference than in younger adults [24–28], a greater need to concentrate on walking due to comorbidities (e.g., after limb loss) [29], and decreased fitness and gait quality [28, 30]. The abovementioned associations are particularly problematic during every day multitasking situations when cognitively demanding tasks are performed while walking and may result in greater risk of falls and injury [31, 32].

The number of falls and/or accidental injuries associated with cellular phone use during walking is growing rapidly [9]. Every year, an estimated 30–40% of general patients over the age of 65 will fall at least once [32, 33]. Falls are one of the major causes of mortality and morbidity in older adults and can lead to moderate to severe injuries, fear of falling, loss of independence, and reduced ability to conduct daily activities. In one-third of these patients, falls can result in death [34]. Falls account for 87% of all fractures in the elderly. One of the major risk factors for falls is impaired balance and gait, as well as cognitive decline, especially attention and executive dysfunction. These data justify the undertaking of investigations into recognizing and better understanding cognitive-motor function. Moreover, these data emphasize the importance of finding methods to improve cognitive-motor function, reduce fall risk, and enhance mobility in adults, including postmenopausal women, who are more vulnerable to bone fractures due to osteoporosis. The cognitive demand of gait control is usually explored with dual-task methodology, in which a single task (walking only) is followed by a dual task (walking while performing a cognitive task) [1, 10, 34]. In such investigations, gait analysis has been done using such methods as GAITRite(r) [35], accelerometry [36, 37], or BTS SMART systems [38]. A BTS SMART system is dedicated to the complex biomechanical analysis of motion and synchronizes and manages kinematic, kinetic, electromyographic, and video data.

The aim of our study was to evaluate the influence of two different demanding cognitive tasks on gait parameters using BTS SMART system analysis. Although there are available several studies concerning the overlap of motor and cognitive function [12–23], according to our best knowledge, such relationships in healthy postmenopausal woman, without cognitive impairment, were not previously investigated. Moreover, in our study, as never before, we performed a comparison of the effect of two different cognitive tasks on gait parameters. Only few publications are also available in which motor functions were examined by means of a BTS SMART system that allows three-dimensional evaluation of gait parameters.

## 2. Material and Methods

### 2.1. Participants

The study comprised 53 postmenopausal women aged 64.5 ± 6.7 years (age range: 47–79 years). Subjects were recruited through advertisements posted on billboards in our outpatient clinic and other health care centers (i.e., in other hospitals and external outpatients clinics in our town), as well as through advertisements in local newspapers. The inclusion criteria were as follows: female gender, menopause, and the ability to undergo an examination using a BTS SMART system. The exclusion criteria were still menstruating, a history of psychiatric, neurological, or somatic illness (including metabolic syndromes, diabetes mellitus, and cancer), substance abuse, or dementia. Individuals with serious neurological and psychiatric disturbances were excluded using a MINI-Plus interview with a cut-off score ≥ 27 [39, 40].

### 2.2. Instruments and Procedure

This study was performed according to a dual-task design: free walking (single task) and two dual-task conditions of walking while performing a cognitive task, each task with a different level of attention demand. Gait tests were conducted with the use of a comprehensive BTS SMART analysis system. The system comprised 16 optoelectronic cameras and two dynamometric platforms. The registration of parameters involved sending infrared rays from optoelectronic lamps towards passive markers placed on the patient's body. The acquired data were transferred to a computer and then, using specialized software, the coordinates of the markers in space were determined. Classical gait parameters are walking speed, stride time and length, step time and length, and the durations of stance phase and swing phase [41]. The asymmetry of gait was also checked through calculation of differences in respective BTS-gait parameters values between left and right foot. However, BTS-SMART system device enables a thorough evaluation of multiple spatial-temporal parameters of gait and we aimed our study to evaluate an effect dual task on the balance and coordination. Therefore, in addition to analyzing the basic gait parameters we have evaluated parameters, which gave such possibility:

1. Foot support phase: the time between heel contact with the ground and a reflection of the fingers.
2. Foot double support phase: the time in which both feet are in contact with the ground.
3. L/R foot support duration: a value measured in seconds and normalized to % to estimate the gait cycle.

Cycles were as follows: four transition paths, eight steps at each stage of the study, for a total minimum of thirty-two steps in the calculation of mean value. Cycles were taken into account, only those who had no technical defects or such, for example, loosen marker.

All parameters were compared in terms of asymmetry between the right and the left lower limb. Quite notable that the asymmetry of gait may occur as a natural process, if the tests of gait affect motor coordination is an asymmetry might increase.

The gait parameters were analyzed three times: during free gait (basic performance), during gait combined with the performance of a simple cognitive task (SCT), and during gait combined with the performance of a complex cognitive task (CCT). During the SCT, subjects were asked to recite the Polish alphabet in the correct order. In the CCT test patients were required to decide in which quoter of the clock is the given time by the researcher in the verbal assignment (e.g., 5 past 12, 10 to 11, 10 past 4, and 20 to 7). The first activity, the SCT, involved the assessment of the performance of a simple automatic memory task, while the second, the CCT, was a more complex activity involving attention, executive functions, and memory. These cognitive tests are based on the references and the experience of the Chair of Clinical Neuropsychology, where our research was conducted [1, 42–44].

Tests were assigned in a randomized order, where the patients selected the tasks in sequences, 1 out of 6 possible combinations:

1. Free gait, SCT, CCT.
2. CCT, SCT, free gait.
3. SCT, free gait, CCT.
4. Free gait, CCT, SCT.
5. CCT, free gait, SCT.
6. SCT, CCT, free gait.

The study was supervised by two assistants and took place in a well-lit and quiet room. Participants in the study performed the tasks in comfortable shoes and at their preferred gait speed.

#### 2.2.1. Bioethics

The study was performed after the Bioethical Commission of the Nicolaus Copernicus University in Toruń, Ludwik Rydygier Collegium Medicum in Bydgoszcz, had given ethical approval (number KB/721/2012). Each patient expressed their consent regarding participation in the study in writing. The study was performed in accordance with the Declaration of Helsinki.

#### 2.2.2. Statistical Analysis

Statistical analysis was carried out with the use of STATISTICA (a data analysis software system) version 10 from StatSoft, Inc. (2011). The distribution of the variables was evaluated by the Shapiro-Wilk test. The results were presented as mean ± 95% confidence interval (CI) or standard deviation (SD). The statistical significance of differences between respective study phases (simple walking and gait with SCT and CCT) was analyzed using the one- or two-factorial ANOVA method with three repetitions and the Fisher's least significant difference post hoc test.

## 3. Results

A total of 53 female participants aged 64.5 ± 6.7 years were included in the analysis. A comparison of gait parameters acquired with the use of the BTS SMART system during free gait (single task) and the dual tasks that consisted of either walking while performing a simultaneous SCT or a simultaneous CCT (the dual motor-cognitive tasks) is presented in Table 1.

Attention allocation during the dual motor-cognitive tasks led to statistically significant results with regard to the following individual quantitative gait variables: extension of the left and right foot gait cycle, shortening of the right leg step length, reduction of left and right leg swing speed, and reduction of gait speed (Table 1). It did not affect the values for the asymmetry of gait parameters. The majority of these changes were statistically significant only when basic performance (free walking) was compared with CCT (Table 1). Complex cognitive task compared to SCT induced greater BTS-gait parameters disturbances in relation to gait cycle duration, step length, and swing speed (Table 1).

**Figure 1:**
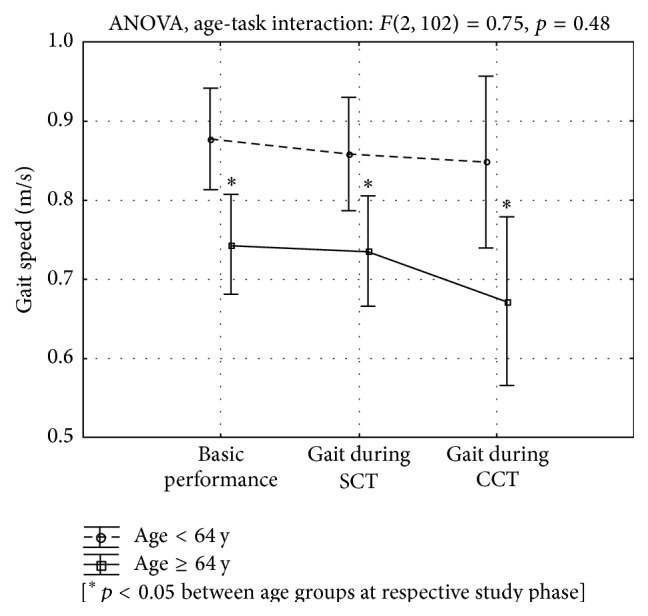
The median age split analysis (<64 y and ≥64 y) of gait speed during basic performance and while performing simple (SCT) and complex cognitive tasks (CCT). ANOVA *F*(2,102) = 0.75; *p* = 0.48.

As the studied group was characterized by a large age range and due to known, age-related feedback effects on gait pattern and cognitive function as presented in Section 1, we performed a split analysis on the median of the subjects' age, which amounted to 64 y. Both age (<64 y or ≥64 y) and the different dual-task stages (basic performance, SCT, and CCT) had a statistically significant main effect which, principally, comprised changes in left and right foot gait cycle duration, right leg step length, left and right leg swing speed, and gait speed. However, the effect of the interaction of these variables was not significant.  Figure 1 illustrates, for instance, the split analysis of the interaction of age (<64 y and ≥64 y) and task performance on gait speed. Older patients had a significantly slower gait than females aged <64 y at every study stage; gait speed was the greatest during basic performance in both age-related groups and in both groups decreased with increase of cognitive task difficultness, in such manner that the course of the lines in both age groups is parallel. This means that both age and dual-task affected gait speed independently.

## 4. Discussion

In this study, quantitative motion analysis using a BTS SMART system was used to assess the effect of a dual task on gait parameters for the purpose of recognizing the clinical importance of the overlap of motor-cognitive functions in postmenopausal woman without significant somatic, neurological, or psychiatric disturbances. We can demonstrate that dual cognitive tasks significantly affected some gait parameters, with a pronounced effect resulting from the level of difficulty of the task (Table 1). Compared to free walking, the performance of SCT and CCT while walking was associated with a statistically significant prolongation of left and right foot gait cycle, shortening of the right leg step, a reduction in left and right leg swing speed, and gait speed (Table 1). These parameters were also independently and significantly affected by the patients' age, as seen in the median split analysis, but the interaction effect of age and task was not statistically significant (Figure 1).

The statistically significant effects of dual-task performance on gait parameters similar to those examined by us have been previously reported, both in healthy people, children, adults, and the elderly, as well as in patients with depression, dementia, past stroke, Parkinson's and Alzheimer's diseases, or multiple sclerosis [3, 12–23, 45, 46]. Theill et al., similarly to us, but in elderly patients with cognitive impairment, demonstrate that counting backwards reduces the speed of gait [47], and Taylor et al. found that, in the same patient group, this dual task reduced gait speed, shortened the step length of the right leg, and prolonged the support and double support phases [48]. Beurskens et al. [49], although in children, observed a significant decrease in gait velocity, stride length, and cadence, as well as an increase in the variability thereof during dual compared to single tasks. In some studies, similarly to ours, performance measures (e.g., cadence) only changed under a high cognitive workload [50]. This demonstrates that an increase in the level of difficulty of cognitive tasks and, consequently, greater engagement of cognitive functions, especially spatial attention and executive function, augment the biomechanical disturbances of gait.

Our observations may have some clinical importance. Firstly, we found that a dual task with a higher level of difficulty and requiring greater attention allocation led to a reduction in gait speed (Figure 1). As gait velocity in older people is recognized as an easy test for detecting risk of cognitive impairment, functional dependence, and state of health [30, 34, 51–53], and a dual task study enabled the early detection of executive function impairment with 89% sensitivity and 87% specificity [8], our observation may show that our subjects have a high probability of an occurrence of cognitive dysfunction in the future. However, confirmation of this discovery of our study needs to be followed up, which would show whether patients like ours might be a target for interventions designed to mitigate functional decline, for example, through training in tai chi-like techniques, which have been shown, in the short term, to reduce cognitive-motor interference and improve balance, gait pattern, and cognitive function [10, 22, 54–57]. On the other hand, our patients achieved a greater gait speed than the frailty-related threshold of 0.6 m/s [46]. Secondly, our findings showed that attention allocation associated with the simultaneous performance of cognitive tasks affects gait pattern, which confirms that gait is not an automatic function but needs cognitive control, and that the performance of an attention-demanding task while walking, for example, the use of a mobile phone, worsens gait quality, which may increase the risk of falls, especially when the walker's attentional capacity is limited [58]. On the other hand, the decline, mainly in the motor performance during the dual task, indicates that people prioritized the cognitive task [59].

Unfortunately, our investigation has some methodological shortcomings, which decrease the strength of our conclusion. The main limitation was the small number in the study group, although it was no smaller than that in the majority of the above-cited studies [19, 20]. As the age of the subjects significantly affects both cognitive and motor function (e.g., gait speed, stride length, and stride time variability) [27, 28, 30, 57, 58, 60], the wide age range of our patients should be recognized as the second study limitation. The correctness of such thinking was confirmed by our split analysis (Figure 1). We also did not measure our patients' performance in the cognitive tests. The fourth limitation of our study is a lack of follow-up, which might show the course and complications of gait impairments revealed during the dual task.

## 5. Conclusions

The dual task significantly affected some gait parameters, with more difficult cognitive tasks and older age having more pronounced effects. This might result from the overlapping of information processing in the central nervous system and may be a cause of increased risk of accidents when, for example, cellular phones are used while walking. Further study with a long follow-up is needed to verify if dual-task performance can be improved by training.

## Figures and Tables

**Table 1:** A comparison of gait parameters in basic conditions and during performance of a simple cognitive task (SCT) and a complex cognitive task (CCT) (*n* = 53).

Gait parameter	Basic performance		SCT		CCT	
	Left foot	Right foot	Left foot	Right foot	Left foot	Right foot
Support phase (s)	0.63	0.63	0.63	0.63	0.63	0.63
	0.62–0.64	0.62–0.64	0.62–0.64	0.62–0.64	0.61–0.64	0.61–0.65

Double support phase (s)	0.13	0.13	0.12	0.14	0.13	0.15
	0.12–0.13	0.12–0.14	0.12–0.13	0.13–0.15	0.12–0.14	0.14–0.17

Support phase duration (s)	0.70	0.70	0.72	0.72	0.75	0.76
	0.67–0.73	0.67–0.73	0.69–0.76	0.68–0.76	0.71–0.79	0.72–0.80

Gait cycle duration (s)	1.11	1.11	1.15^*∗*^	1.13	1.19^*∗*#^	1.19^*∗*#^
	1.07–1.15	1.07–1.15	1.09–1.20	1.08–1.18	1.14–1.24	1.13–1.24

Step length (m)	0.50	0.49	0.50	0.49	0.49	0.47^*∗*#^
	0.48–0.52	0.47–0.51	0.48–0.52	0.47–0.51	0.45–0.52	0.45–0.49

Step width (m)	0.15		0.18		0.16	
	0.149–0.160		0.13–0.24		0.15–0.16	

Swing speed	2.30	2.31	2.26	2.27	2.04^*∗*#^	2.12^*∗*#^
	2.17–2.42	2.19–2.43	2.12–2.39	2.14–2.40	1.91–2.17	1.99–2.25

Gait speed (m*∗*s^−1^)	0.81		0.80		0.76^*∗*^	
	0.76–0.86		0.74–0.85		0.68–0.84	

Data presented as mean ± 95% CI.

Differences versus basic performance significance ^*∗*^ *p* < 0.05.

Differences SCT versus CCT significance ^#^ *p* < 0.05.

## References

[1] Nascimbeni A., Caruso S., Salatino A. (2015). Dual task-related gait changes in patients with mild cognitive impairment. *Functional Neurology*.

[2] Al-Yahya E., Dawes H., Smith L., Dennis A., Howells K., Cockburn J. (2011). Cognitive motor interference while walking: a systematic review and meta-analysis. *Neuroscience and Biobehavioral Reviews*.

[3] Ble A., Volpato S., Zuliani G. (2005). Executive function correlates with walking speed in older persons: the InCHIANTI study. *Journal of the American Geriatrics Society*.

[4] Beauchet O., Launay C. P., Fantino B., Annweiler C., Allali G. (2015). Episodic memory and executive function impairments in non-demented older adults: which are the respective and combined effects on gait performances?. *Age*.

[5] Bruder G., Lubas P., Steinicke F. (2015). Cognitive resource demands of redirected walking. *IEEE Transactions on Visualization and Computer Graphics*.

[6] Licence S., Smith R., McGuigan M. P., Earnest C. P. (2015). Gait pattern alterations during walking, texting and walking and texting during cognitively distractive tasks while negotiating common pedestrian obstacles. *PLOS ONE*.

[7] Lim J., Amado A., Sheehan L., Van Emmerik R. E. A. (2015). Dual task interference during walking: the effects of texting on situational awareness and gait stability. *Gait & Posture*.

[8] Perrochon A., Kemoun G., Watelain E., Dugué B., Berthoz A. (2015). The ‘Stroop Walking Task’: an innovative dual-task for the early detection of executive function impairment. *Neurophysiologie Clinique*.

[9] Kao P. C., Higginson C. I., Seymour K., Kamerdze M., Higginson J. S. (2015). Walking stability during cell phone use in healthy adults. *Gait & Posture*.

[10] Verghese J., Ayers E., Barzilai N. (2014). Motoric cognitive risk syndrome: multicenter incidence study. *Neurology*.

[11] Allali G., Ayers E. I., Verghese J. (2016). Motoric cognitive risk syndrome subtypes and cognitive profiles. *The Journals of Gerontology A: Biological Sciences and Medical Sciences*.

[12] Michalak J., Troje N. F., Fischer J., Vollmar P., Heidenreich T., Schulte D. (2009). Embodiment of sadness and depression-gait patterns associated with dysphoric mood. *Psychosomatic Medicine*.

[13] Wright S. L., Kay R. E., Avery E. T., Giordani B., Alexander N. B. (2011). The impact of depression on dual tasking among patients with high fall risk. *Journal of Geriatric Psychiatry and Neurology*.

[14] Gabel N. M., Crane N. A., Avery E. T. (2015). Dual-tasking gait variability and cognition in late-life depression. *International Journal of Geriatric Psychiatry*.

[15] Bruce-Keller A. J., Brouillette R. M., Tudor-Locke C. (2012). Relationship between cognitive domains, physical performance, and gait in elderly and demented subjects. *Journal of Alzheimer's Disease*.

[16] Hausdorff J. M., Balash J., Giladi N. (2003). Effects of cognitive challenge on gait variability in patients with Parkinson's disease. *Journal of Geriatric Psychiatry and Neurology*.

[17] Smulders K., Esselink R. A. J., Weiss A., Kessels R. P. C., Geurts A. C. H., Bloem B. R. (2012). Assessment of dual tasking has no clinical value for fall prediction in Parkinson's disease. *Journal of Neurology*.

[18] Peterson D. S., King L. A., Cohen R. G., Horak F. B. Cognitive contributions to freezing of gait in Parkinson disease: implications for physical rehabilitation. *Physical Therapy*.

[19] Wang X. Q., Pi Y. L., Chen B. L., Wang R., Li X., Chen P. J. (2015). Cognitive motor intervention for gait and balance in Parkinson's disease: systematic review and meta-analysis. *Clinical Rehabilitation*.

[20] Tillman A., Muthalib M., Hendy A. M. (2015). Lower limb progressive resistance training improves leg strength but not gait speed or balance in Parkinson's disease: a systematic review and meta-analysis. *Frontiers in Aging Neuroscience*.

[21] Fernández-Lago H., Bello O., López-Alonso V., Sánchez J. A., Morenilla L., Fernández-del-Olmo M. Á. (2015). Gait pattern and cognitive performance during treadmill walking in Parkinson disease. *American Journal of Physical Medicine & Rehabilitation*.

[22] Maquet D., Lekeu F., Warzee E. (2010). Gait analysis in elderly adult patients with mild cognitive impairment and patients with mild Alzheimer's disease: simple versus dual task: a preliminary report. *Clinical Physiology and Functional Imaging*.

[23] Wajda D. A., Sosnoff J. J. (2015). Cognitive-motor interference in multiple sclerosis: a systematic review of evidence, correlates, and consequences. *BioMed Research International*.

[24] Harada T., Miyai I., Suzuki M., Kubota K. (2009). Gait capacity affects cortical activation patterns related to speed control in the elderly. *Experimental Brain Research*.

[25] Verghese J., Kuslansky G., Holtzer R. (2007). Walking while talking, effect of task prioritization in the elderly. *Archives of Physical Medicine and Rehabilitation*.

[26] Malcolm B. R., Foxe J. J., Butler J. S., De Sanctis P. (2015). The aging brain shows less flexible reallocation of cognitive resources during dual-task walking: a mobile brain/body imaging (MoBI) study. *NeuroImage*.

[27] Stöckel T., Jacksteit R., Behrens M., Skripitz R., Bader R., Mau-Moeller A. (2015). The mental representation of the human gait in young and older adults. *Frontiers in Psychology*.

[28] Mirelman A., Bernad-Elazari H., Nobel T. (2015). Effects of aging on arm swing during gait: the role of gait speed and dual tasking. *PLoS ONE*.

[29] Morgan S. J., Hafner B. J., Kelly V. E. (2015). The effects of a concurrent task on walking in persons with transfemoral amputation compared to persons without limb loss. *Prosthetics and Orthotics International*.

[30] Garcia-Pinillos F., Cozar-Barba M., Munoz-Jimenez M., Soto-Hermoso V., Latorre-Roman P. (2015). Gait speed in older people: an easy test for detecting cognitive impairment, functional independence, and health state. *Psychogeriatrics*.

[31] Bridenbaugh S. A., Kressig R. W. (2015). Motor cognitive dual tasking: early detection of gait impairment, fall risk and cognitive decline. *Zeitschrift für Gerontologie und Geriatrie*.

[32] Ambrose A. F., Cruz L., Paul G. (2015). Falls and fractures: a systematic approach to screening and prevention. *Maturitas*.

[33] Davison J., Bond J., Dawson P., Steen I. N., Kenny R. A. (2005). Patients with recurrent falls attending Accident & Emergency benefit from multifactorial intervention—a randomised controlled trial. *Age and Ageing*.

[34] Makizako H., Shimada H., Doi T. (2015). Cognitive functioning and walking speed in older adults as predictors of limitations in self-reported instrumental activity of daily living: prospective findings from the Obu Study of Health Promotion for the Elderly. *International Journal of Environmental Research and Public Health*.

[35] Guedes R. C., Dias R. C., Pereira L. S. M., Silva S. L. A., Lustosa L. P., Dias J. M. D. (2014). Influence of dual task and frailty on gait parameters of older community-dwelling individuals. *Brazilian Journal of Physical Therapy*.

[36] Yoneyama M., Mitoma H., Higuma M., Sanjo N., Yokota T., Terashi H. (2015). Ambulatory gait behavior in patients with dementia: a comparison with Parkinson’s disease. *IEEE Transactions on Neural Systems and Rehabilitation Engineering*.

[37] Gillain S., Dramé M., Lekeu F. (2015). Gait speed or gait variability, which one to use as a marker of risk to develop Alzheimer disease? A pilot study. *Aging Clinical and Experimental Research*.

[38] Dziuba A. K., Żurek G., Garrard I., Wierzbicka-Damska I. (2015). Biomechanical parameters in lower limbs during natural walking and Nordic walking at different speeds. *Acta of Bioengineering and Biomechanics*.

[39] Tombaugh T. N., McIntyre N. J. (1992). The mini-mental state examination: a comprehensive review. *Journal of the American Geriatrics Society*.

[40] Tombaugh T. N. (2005). Test-retest reliable coefficients and 5-year change scores for the MMSE and 3MS. *Archives of Clinical Neuropsychology*.

[41] Senden R., Grimm B., Heyligers I. C., Savelberg H. H. C. M., Meijer K. (2009). Acceleration-based gait test for healthy subjects: reliability and reference data. *Gait and Posture*.

[42] Qu X. (2014). Age-related cognitive task effects on gait characteristics: do different working memory components make a difference?. *Journal of NeuroEngineering and Rehabilitation*.

[43] Borkowska A. (2009). Znaczenie zaburzeń funkcji poznawczych i możliwości ich oceny w chorobach psychicznych. *Psychiatria w Praktyce Klinicznej*.

[44] Jaracz M., Bieliński M., Junik R. (2009). Zaburzenia pamięci operacyjnej i funkcji wykonawczych u osób z patologiczną otyłością. *Psychiatria*.

[45] Yogev-Seligmann G., Rotem-Galili Y., Mirelman A., Dickstein R., Giladi N., Hausdorff J. M. (2010). How does explicit prioritization alter walking during dual-task performance? Effects of age and sex on gait speed and variability. *Physical Therapy*.

[46] Stephan Y., Sutin A. R., Terracciano A. (2015). ‘Feeling younger, walking faster’: subjective age and walking speed in older adults. *Age*.

[47] Theill N., Martin M., Schumacher V., Bridenbaugh S. A., Kressig R. W. (2011). Simultaneously measuring gait and cognitive performance in cognitively healthy and cognitively impaired older adults: the basel motor-cognition dual-task paradigm. *Journal of the American Geriatrics Society*.

[48] Taylor M. E., Delbaere K., Mikolaizak A. S., Lord S. R., Close J. C. T. (2013). Gait parameter risk factors for falls under simple and dual task conditions in cognitively impaired older people. *Gait and Posture*.

[49] Beurskens R., Muehlbauer T., Granacher U. (2015). Association of dual-task walking performance and leg muscle quality in healthy children. *BMC Pediatrics*.

[50] Knaepen K., Marusic U., Crea S. (2015). Psychophysiological response to cognitive workload during symmetrical, asymmetrical and dual-task walking. *Human Movement Science*.

[51] Ojagbemi A., D'Este C., Verdes E., Chatterji S., Gureje O. (2015). Gait speed and cognitive decline over 2 years in the Ibadan study of aging. *Gait & Posture*.

[52] Wrightson J. G., Ross E. Z., Smeeton N. J. (2015). The effect of cognitive task type and walking speed on dual-task gait in healthy adults. *Motor Control*.

[53] Basile G., Catalano A., Mandraffino G. (2015). Cognitive impairment and slow gait speed in elderly outpatients with arterial hypertension: the effect of blood pressure values. *Journal of the American Geriatrics Society*.

[54] Stothart C. R., Simons D. J., Boot W. R., Kramer A. F. (2014). Is the effect of aerobic exercise on cognition a placebo effect?. *PLoS ONE*.

[55] Sun J., Kanagawa K., Sasaki J., Ooki S., Xu H., Wang L. (2015). Tai chi improves cognitive and physical function in the elderly: a randomized controlled trial. *Journal of Physical Therapy Science*.

[56] Fritz N. E., Cheek F. M., Nichols-Larsen D. S. (2015). Motor-cognitive dual-task training in persons with neurologic disorders: a systematic review. *Journal of Neurologic Physical Therapy*.

[57] Nishiguchi S., Yamada M., Tanigawa T. (2015). A 12-week physical and cognitive exercise program can improve cognitive function and neural efficiency in community-dwelling older adults: a randomized controlled trial. *Journal of the American Geriatrics Society*.

[58] Mazaheri M., Hoogkamer W., Potocanac Z. (2015). Effects of aging and dual tasking on step adjustments to perturbations in visually cued walking. *Experimental Brain Research*.

[59] Agmon M., Kodesh E., Kizony R. (2014). The effect of different types of walking on dual-task performance and task prioritization among community-dwelling older adults. *The Scientific World Journal*.

[60] Doi T., Shimada H., Makizako H. (2015). Mild cognitive impairment, slow gait, and risk of disability: a prospective study. *Journal of the American Medical Directors Association*.

